# A Prospective Cohort Study Investigating Associations between Hyperemesis Gravidarum and Cognitive, Behavioural and Emotional Well-Being in Pregnancy

**DOI:** 10.1371/journal.pone.0027678

**Published:** 2011-11-18

**Authors:** Fergus P. McCarthy, Ali S. Khashan, Robyn A. North, Rona Moss-Morris, Philip N. Baker, Gus Dekker, Lucilla Poston, Louise C. Kenny, on behalf of the SCOPE consortium

**Affiliations:** 1 Department of Obstetrics and Gynecology, The Anu Research Centre, Cork University Maternity Hospital, University College Cork, Cork, Ireland; 2 Division of Reproduction and Endocrinology, King's College London, London, United Kingdom; 3 School of Psychology, University of Southampton, Southampton, United Kingdom; 4 University of Alberta, Edmonton, Canada; 5 The Royal Alex Hospital, Edmonton, Canada; 6 University of Manchester, Manchester, United Kingdom; 7 Women's and Children's Division Lyell McEwin Hospital, University of Adelaide, Adelaide, South Australia; Institute of Zoology, Chinese Academy of Sciences, China

## Abstract

**Objectives:**

To investigate the association between hyperemesis gravidarum and altered cognitive, behavioural and emotional well-being in pregnancy.

**Methods:**

The study cohort consisted of 3423 nulliparous women recruited in the Screening for Pregnancy Endpoints (SCOPE) study performed in Auckland, New Zealand; Adelaide, Australia; Cork, Ireland; Manchester and London, United Kingdom between November 2004 and August 2008. Women were interviewed at 15±1 weeks' gestation and at 20±1weeks' gestation. Women with a diagnosis of hyperemesis gravidarum (HG) were compared with women who did not have a diagnosis of HG. Main outcome measures included the Short form State- Trait Anxiety Inventory (STAI) score (range 6–24), Perceived Stress Scale score (PSS, range 0–30), Edinburgh Postnatal Depression Scale (EPDS) score (range 0–30 or categories a–c) and behavioural responses to pregnancy score (limiting/resting [range 0–20] and all-or-nothing [range 0–28]).

**Results:**

During the study period 164 women suffered from HG prior to their 15 week interview. Women with HG had significantly higher mean STAI, PSS, EPDS and limiting response to pregnancy scores compared to women without HG. These differences were observed at both 15±1 and 20±1 weeks' of gestation. The magnitude of these differences was greater in women with severe HG compared to all women with HG. Women with severe HG had an increased risk of having a spontaneous preterm birth compared with women without HG (adjusted OR 2.6 [95% C.I. 1.2, 5.7]).

**Conclusion:**

This is the first large prospective study on women with HG. Women with HG, particularly severe HG, are at increased risk of cognitive, behavioural and emotional dysfunction in pregnancy. Women with severe HG had a higher rate of spontaneous preterm birth compared to women without HG. Further research is required to determine whether the provision of emotional support for women with HG is beneficial.

## Introduction

Up to 80% of all pregnant women experience some form of nausea and vomiting during their pregnancy [1], [2], [3]. Hyperemesis gravidarum (HG) is persistent and excessive vomiting starting before the end of the 22nd week of gestation [4]. Affecting approximately 0.3–2.0% of pregnancies, HG is the commonest indication for admission to hospital in the first half of pregnancy and is second only to preterm labour as a cause of hospitalisation during pregnancy [5], [6], [7]. Although HG has an unknown aetiology, research has demonstrated that HG is transmitted from mothers to daughters suggesting a strong influence of maternal genes and/or environment [8].

The psychological implication of illness in pregnancy is poorly understood. Studies investigating the association between HG, pregnancy outcomes and maternal psychological morbidity have provided conflicting results [7], [9]. The data have been limited by retrospective study design [10], [11], [12], [13], small numbers [14], bias and variable definitions of HG [13], [15], [16]. HG has been reported to be associated with an increased risk of adverse pregnancy outcomes such as preterm birth and small for gestational age infants (SGA) [10], [11], [17]. Female infant sex has also been associated with HG [6], [18], [19], [20]. A recent study demonstrated that adverse outcomes occurring in pregnancies affected by HG are explained in part by the differences in maternal characteristics between woman with and without HG [17]. Antenatal stress, anxiety and depression are independently associated with adverse pregnancy outcomes such as low birth weight and preterm birth [21], [22], [23].

In a large prospective cohort of nulliparous women with a singleton pregnancy, we investigated the association between HG and cognitive, behavioural and emotional well-being and determined whether the severity of HG influenced any relationship observed. The study also aimed to clarify if HG is associated with adverse pregnancy outcomes.

## Methods

SCOPE (Screening for Pregnancy Endpoints) is a prospective, multicentre cohort study with the main aim of developing screening tests to predict pre-eclampsia, SGA infants, and spontaneous preterm birth [24]. Participants were healthy nulliparous women with singleton pregnancies recruited between November 2004 and August 2008 in Auckland, New Zealand, Adelaide, Australia, Cork, Ireland, and Manchester and London, United Kingdom.

Women were recruited at 15±1 weeks' gestation through hospital antenatal clinics, obstetricians, general practitioners, community midwives, and self referral in response to advertisements or recommendations of friends, as previously described [25]. Women were excluded if they were considered to be at high risk of pre-eclampsia, small for gestational age babies, or spontaneous preterm birth because of underlying medical conditions, gynaecological history, three or more previous miscarriages, three or more terminations of pregnancy, or had received interventions, such as aspirin, that might modify pregnancy outcome [24].

SCOPE participants were interviewed and examined by SCOPE research midwives at 15±1 and 20±1 weeks' gestation. Data were entered at the time of interview into an internet accessed, auditable database developed by Medscinet AB, Sweden. Participants were followed up prospectively, with pregnancy outcome data collected by research midwives. Each participant's data was individually checked (including for any data entry errors in the lifestyle questionnaire) and using a customised software program to detect any systematic data entry errors. At 15±1 and 20±1 weeks' gestation, participants were questioned regarding vomiting in pregnancy, gestation of onset and cessation of vomiting and whether this vomiting resulted in hospital admission, IV fluids, nasogastric feeding or weight loss.

Primary outcomes reported at 15±1 week and 20±1 week interview were anxious mood measured using the short form of the State Trait Anxiety Index (STAI) [26], how much stress the individual feels they are currently experiencing measured using the Perceived Stress Scale (PSS) [27], depressed mood measured using the Edinburgh Postnatal Depression Scale (EPDS) [28], [29] and pregnancy related behaviour measured using a behavioural response to pregnancy scale [30] (Table 1). All-or-nothing behaviour describes a pattern of alternating extremes of behaviour characterised by a cyclical response of pushing oneself to keep going until this no longer feels physically possible. Limiting/resting behaviour refers to a tendency to curtail activities of daily living in response to symptoms or to respond to symptoms by resting, e.g. “I have avoided my usual activities”. The EPDS were calculated using both EPDS as a continuous variable and as a categorical variable with 3 categories as described in Table 1.

**Table 1:** Cognitive, behavioural and emotional health scores and their interpretations.

Psychological and behavioural scales	Score range and interpretation
Edinburgh Postnatal Depression Scale (EPDS) [29]	As a continuous measure (0–30), where a higher score indicates a higher probability of depression
	OR
	As a categorical variable with the following 3 categories
	1.EPDS <5: unlikely to experience depression post partum
	2.EPDS 5–9: increased risk of depression in the next year
	3.EPDS >9: very likely depressed
	0–40, with high scores representing higher perceived stress
Perceived Stress Scale [27]	Two subscales:
	1.Limiting/resting behaviour (0–20)
	2.All-or-Nothing behaviour (0–28)
Behavioural Responses to Pregnancy (adapted from the Behavioural Response to Illness Questionnaire [30])	6–24, with high scores indicating high state anxiety
Short form State-Trait Anxiety Inventory [26]	

Secondary outcomes included spontaneous preterm birth, pre-eclampsia, birthweight, SGA and infant sex ratio.

HG was defined as repeated vomiting in early pregnancy not due to other causes (e.g., gastroenteritis) requiring any of the following: inpatient admission, day stay with IV fluids, nasogastric feeding (at home or in hospital) or vomiting associated with loss of >5% of her booking weight. Women with hospitalized HG were considered as having severe HG.

The estimated date of delivery was calculated from a certain last menstrual period date. The estimated date of delivery was only adjusted if either a scan at less than 16 weeks' gestation found a difference of seven or more days between the scan gestation and that calculated by the last menstrual period, or at a 20 week scan a difference of 10 or more days was found between the scan gestation and that calculated from the last menstrual period. If the last menstrual period date was uncertain, scan dates were used to calculate the estimated date of delivery. SGA was defined as birthweight below the 10^th^ customised centile adjusted for maternal weight, height, parity, ethnic group, and infant sex (www.gestation.net). [31] Spontaneous preterm birth was defined as spontaneous preterm labour or preterm premature rupture of the membranes resulting in preterm birth at less than 37 weeks' gestation. Pre-eclampsia was defined as systolic blood pressure ≥140 mmHg and/or diastolic blood pressure ≥90 mmHg on at least two occasions four hours apart after 20 weeks' gestation but before the onset of labour or postpartum, with proteinuria (24 hour urinary protein ≥300 mg, or spot urine protein to creatinine ratio ≥30 mg/mmol creatinine, or urine dipstick protein ≥2+) or any multisystem complication of pre-eclampsia [32].

Student's t-test was used to compare continuous variables and χ^2^ was used to compare categorical variables between women with HG and those without HG. Logistic regression and linear regression with robust variance estimation were used to analyse the binary and continuous outcome measures respectively. Each of the primary outcomes was analysed as a continuous variable with the exception of the EPDS which was also analysed as a categorical variable using ordered logistic regression. All analyses were adjusted for the potential confounding effects of maternal age, smoking, alcohol consumption, ethnicity, body mass index (BMI), marital status, SCOPE centre and job status as listed in Table 2. The regression models for birthweight, SGA and spontaneous preterm birth were also examined for other potential confounders including folic acid intake, multivitamin use, vaginal bleeding in pregnancy, miscarriage, termination of pregnancy and large loop excision of the cervical transformation zone (LLETZ). None of these variables had a significant effect on the models and were, therefore, not included in the final analysis.

**Table 2:** Characteristics of participants by HG status at 15 weeks' gestation.

	Hyperemesis Gravidarum(n = 164)	No Hyperemesis Gravidarum(n = 3259)	P value
Age (years)	25.8 (5.7)	28.3 (5.8)	<0.001
Ethnic origin			
Caucasian	132 (81)	2850 (88)	<0.001
Asian	3 (2)	144 (4)	
Indian	11 (7)	83 (3)	
Polynesian	11 (7)	94 (3)	
Other (including African)	7 (4)	88 (3)	
Married/defacto	152 (93)	2997 (92)	0.74
Single	12 (7)	262 (8)	
Schooling ≤12 years	77 (47)	1544 (47)	0.91
Socioeconomic Index	41 (15)	41 (17)	0.42
Full/part time work	141 (86)	2719 (83)	0.39
No paid work	23 (14)	540 (17)	
Body Mass index (kg/m^2^)			
<20.0	16 (10)	252 (8)	0.82
20–24.9	76 (46)	1561 (48)	
25.0–29.9	45 (27)	914 (28)	
≥30.0	27 (17)	532 (16)	
Miscarriage			
0	145 (88)	2811 (86)	0.28
≥1	19 (12)	448 (14)	
Termination of pregnancy			
0	146 (89)	2810 (86)	0.57
1	15 (9)	386 (12)	
≥2	3 (2)	63 (2)	
LLETZ treatment	9 (6)	125 (4)	0.29
Vaginal bleeding	40 (24)	689 (21)	0.32
Alcohol*	4 (2)	228 (7)	0.02
Smoking*	20 (12)	355 (11)	0.67
Folic Acid*	155 (95)	3069 (94)	0.86
Multivitamins*	86 (52)	1820 (56)	0.60
Recreational drug use*	8 (5)	55 (2)	0.003

Data are mean (SD) or number %.

LLETZ = large loop excision of transformation zone.

P values are for comparisons between the two groups using student t-test or χ2 test , P<0.05.

*At 15±1 week.

We performed two comparisons for **each** outcome measure;

1. The risk of the outcome measure in women with HG (mild+severe) compared to women with no HG (reference group);
2. The risk of the outcome measure in women with severe HG compared to women with no HG. This later comparison was performed by using a three category HG variable; 1) no HG, 2) mild HG and 3) severe HG with no HG as the reference group.

It was unclear whether women with HG had higher cognitive, behavioural and emotional dysfunction scores secondary to vomiting or were predisposed to higher scores which made them vulnerable to developing HG. To investigate this, we compared the risk of the outcome measures in women with a diagnosis of HG prior to the 15 week interview but whose vomiting had ceased before the visit to women with a diagnosis of HG prior to the 15 week interview but with ongoing vomiting continuing at the visit and between the 15 and 20 week interviews. This latter comparison was performed by using a four category HG variable on data from the 20 week interview; 1) no HG, 2) HG before 15 weeks, vomiting ceased before 15 weeks 3) HG before 15 w visit, continuing at 15 w visit, but no vomiting after 15 week visit 4) HG before 15 weeks and ongoing vomiting from 15±1 to 20±1 weeks.

Ethical approval was obtained from local ethics committees [New Zealand; Auckland Ethics committee (AKX/02/00/364), Australia; Central Northern Adelaide Health Service Ethics of Human Research Committee (REC 1712/5/2008), London and Manchester; National Research Ethics Committee, South East Research Ethics committee (06/MRE01/98) and Cork; Clinical Research Ethics Committee of the Cork Teaching Hospital (ECM5(10)05/02/08)] and all women provided written informed consent.

## Results

**Figure 1:**
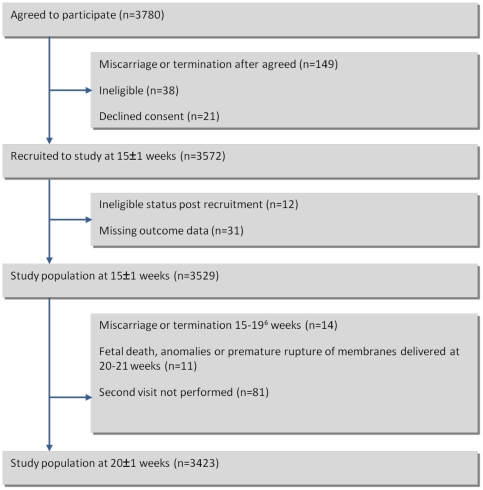
Participants recruited.

3572 women were recruited to the SCOPE study during the study period. Women were excluded from this study if they had incomplete data on cognitive, behavioural and emotional measures at both first and second visits (n = 81). In total, 149 women were excluded from the analysis as described in figure 1. Among the 3423 women in the final study population, 164 (4.8%) had HG, of whom 71 (2.1%) had severe HG requiring hospitalisation. Of the 164 women who had suffered from HG, 74 (45%) had ceased vomiting before 15±1 weeks, 54 (33%) had vomiting at 15±1, but no vomiting after this and 31 (19%) had ongoing vomiting from 15 to 20weeks.

Women with HG were younger, more commonly non Caucasian and less likely to drink alcohol (table 2). BMI, smoking and socioeconomic status did not differ significantly between women with HG and those without (table 2).

At 15±1 and 20±1 weeks' gestation, women with HG had higher mean STAI, PSS, EPDS and limiting/resting responses to pregnancy scores compared with women without HG (table 3). Similarly, women with HG (all HG) had over twice the odds of a higher EPDS categorical score. The magnitude of differences in STAI, PSS, EPDS and limiting response to pregnancy mean scores was greater in women with severe HG than that seen in all women with HG (table 3).

**Table 3:** Association between HG and primary outcomes.

Scale	Gestation measured (weeks)	All HG (n = 164)	All HG (n = 164)	Severe HG (n = 71)	Severe HG (n = 71)
		Unadjusted mean difference (95% CI)	Adjusted mean difference (95% CI)	Unadjusted mean difference (95% CI)	Adjusted mean difference (95% CI)
State Trait Anxiety*	15	4.3 (2.3, 6.3)	3.9 (1.9, 5.9)	5.7 (2.5, 8.9)	4.8 (1.7, 8.0)
	20	2.7 (0.8, 4.5)	2.4 (0.6, 4.2)	4.7 (1.9, 7.4)	4.0 (1.3, 6.7)
Perceived Stress Scale*	15	2.1 (1.0, 3.2)	1.8 (0.7, 2.9)	3.8 (2.3, 5.3)	3.3 (1.7, 4.8)
	20	1.5 (0.5, 2.6)	1.2 (0.1, 2.2)	3.4 (1.8, 5.0)	2.8 (1.2, 4.4)
EPDS*(continuous)	15	2.0 (1.2, 2.8)	1.8 (1.0, 2.6)	3.1 (1.8, 4.4)	2.7 (1.4, 4.0)
	20	1.6 (0.8, 2.3)	1.4 (0.6, 2.1)	2.6 (1.3, 3.8)	2.2(1.0, 3.4)
EPDS**(categorical,OR)	15	2.2 (1.6,2.9)	2.1 (1.6, 2.9)	3.0 (1.9, 4.9)	2.7 (1.6, 4.5)
	20	1.6 (1.1, 2.2)	1.6 (1.2, 2.3)	2.5 (1.6, 4.0)	2.2 (1.3, 3.6)
Limiting response*	15	1.8 (1.3, 2.4)	2.2 (1.5, 2.8)	2.6 (1.7, 3.5)	2.7 (1.8, 3.6)
	20	1.2 (0.6, 1.8)	1.2 (0.6, 1.8)	1.8 (0.8, 2.7)	1.8 (0.8, 2.7)
All or nothing response*	15	−0.1 (−0.8, 0.5)	−0.1 (−0.8, 0.5)	0.4 (−0.6, 1.3)	0.4 (−0.6, 1.4)
	20	0.0 (−0.7, 0.8)	0.0 (−0.8, 0.7)	0.3 (−0.9, 1.5)	0.2 (−0.9, 1.4)

*Score variables are analysed using linear regression with robust estimation.

**Edinburgh Postnatal Depression Score categorical variable presented as odds ratio and calculated using ordered logistic regression.

Models are adjusted for maternal age, smoking, alcohol, ethnic origin, BMI, marital status, SCOPE centre and job status.

Subgroup analysis, shown in table 4, suggests that the elevated stress, depression and limiting response to pregnancy scores occurs secondary to the HG and normalise when the HG improves, although this effect may take weeks to occur.

**Table 4:** Subgroup analysis; Association of primary outcomes at 20 weeks between HG with ongoing vomiting and HG with no ongoing vomiting.

	HG before 15 weeks, ceased by 15 weeks (n = 74)	HG up to 15 weeks, but then ceased (n = 54)	HG before 15 weeks with ongoing vomiting from 15 to 20 weeks (n = 31)
State Trait Anxiety*	3.1 (0.2, 6.1)	0.9 (−1.8, 3.7)	3.3 (−0.6, 7.3)
Perceived Stress Scale*	1.0, (−0.6, 2.6)	0.8 (−0.6, 2.3)	2.3 (−0.6, 5.1)
EPDS* (continuous)	1.0 (−0.2, 2.1)	1.6 (0.3, 2.9)	2.2 (0.5, 3.9)
EPDS**(categorical, OR)	1.3 (0.8, 2.0)	2.1 (1.2, 3.5)	2.4 (1.1, 5.1)
Limiting response*	0.6 (−0.3, 1.5)	1.4 (0.4, 2.5)	2.3 (0.9, 3.7)
All or nothing response*	−0.2 (−1.3, 1.0)	−0.2 (−1.5, 1.0)	0.8 (−0.7, 2.4)

*Score variables are presented as adjusted mean differences (95% CI) from data at 20+ visit, analysed using linear regression with robust estimation.

**Edinburgh Postnatal Depression Score categorical variable presented as odds ratio and calculated using ordered logistic regression.

Models were adjusted for maternal age, smoking, alcohol, ethnic origin, BMI, marital status, SCOPE centre and job status.

N = 5 excluded due to small numbers. These were women with HG before 15 w visit, ceased before 15 w visit, restarted vomiting between 15+ and 20+ visits.

In contrast, more than five weeks following the cessation of vomiting, anxiety scores remain elevated in women with HG (adjusted mean difference 3.1 [95% C.I 0.2, 6.1]).

Overall, women with HG were not more likely to have a spontaneous preterm birth whereas women with severe HG had an increased risk of spontaneous preterm birth compared with women without HG (adjusted OR 2.6 [95% C.I. 1.2, 5.7). Nine percent of women with HG (15 of 164) developed pre-eclampsia compared to 5% of the 3259 women without HG but after adjustment for confounders this increase was not significant (adjusted OR 1.5 [95% C.I. 0.9, 2.7). No differences in other secondary outcomes (SGA, sex ratio and birthweight) were detected (Table 5).

**Table 5:** Association between HG and secondary outcomes.

	All HG (n = 164)	All HG (n = 164)	Severe HG (n = 71)	Severe HG (n = 71)
	Estimate (95% CI)	Adjusted estimate (95% CI)*	Estimate (95% CI)	Adjusted estimate (95% CI)*
Spontaneous preterm birth*	1.6 (0.9, 2.9)	1.6 (0.9, 3.0)	2.6 (1.1, 5.5)	2.6(1.2, 5.7)
Pre-eclampsia*	1.9 (1.1, 3.2)	1.5 (0.9, 2.7)	2.3 (1.1, 4.9)	1.9 (0.9, 4.1)
Small for gestational age*	1.0 (0.6, 1.7)	1.0 (0.6, 1.7)	0.8 (0.3, 1.8)	0.7 (0.3, 1.7)
Birthweight (g)**	−50 (−156, 56)	−30 (−133, 73)	−127 (−308, 54)	−87 (−259, 84)
Infant sex ratio* (male:female)	1.06 (0.77, 1.45)	1.00 (0.74, 1.38)	1.12 (0.70, 1.79)	1.06 (0.67, 1.69)

*Analysed using logistic regression and presented as adjusted odds ratios (95% CI).

**Analysed using linear regression with robust estimation and presented as adjusted mean differences (95% CI).

All regression models were adjusted for maternal age, smoking, alcohol, ethnic origin, infant sex, SCOPE centre and BMI.

## Discussion

This is the first large prospective cohort study of healthy nulliparous women with HG which has demonstrated that women with HG displayed elevated scores for anxiety, stress, depression and limiting behaviour in response to pregnancy. The magnitude of these scores was greatest in women with severe HG. The elevated scores observed in stress, depression and limiting behaviour in response to pregnancy appeared to be related to the vomiting associated with HG and resolution of these scores occurs several weeks following the cessation of vomiting.

In contrast, anxiety scores remained elevated over the five weeks following the cessation of vomiting. This may suggest that women with HG may be more trait anxious and that anxiety may contribute to the onset of the symptoms. Alternatively, anxiety associated with HG may take much longer than depression and stress scores to resolve.

Despite stress, depression and limiting behaviour scores normalising several weeks following the cessation of vomiting, severe HG was associated with long term adverse pregnancy outcomes, having nearly three times the odds of a spontaneous preterm birth. This increased rate of preterm birth may have occurred secondary to an elevated state of anxiety. Alternatively it may reflect increased stress hormones, such as maternal cortisol and catecholamines, in response to the HG resulting in raised corticotrophin releasing hormone which has been implicated in preterm birth [33], [34].

The strengths of our study are that detailed information about cognitive, behavioural and emotional health in pregnancy was collected prospectively and pregnancy outcome data were available in more than 99% of participants. Pregnancy outcome was assigned according to pre-specified criteria and stringent data monitoring protocols ensured the quality of the data.

A limitation is the use of self reported scales and questionnaires as indicators of depression etc rather than a clinical diagnosis. The diagnosis of HG was recorded at 15 weeks' gestation which may have resulted in recall bias for HG occurring earlier in the pregnancy. Given the stringent definition of HG, recall bias is likely to be minimal. Data on cognitive, behavioural and emotional health in women was collected within approximately ten weeks of HG. Unfortunately these data were not available in the first trimester, after 20 weeks' of gestation or outside of pregnancy. Consequently, despite conducting a subgroup analysis related to vomiting status, interpretation of any potential causal effect of HG is guarded.

Published data regarding the association between HG and psychological well-being and adverse pregnancy outcomes are limited with conflicting results, possibly due to the wide range of definitions used for HG and nausea and vomiting of pregnancy Some studies have concluded that HG is associated with psychiatric morbidity or co-existent psychiatric illness or a negative psychosocial impact [35], [36], [37]. Others refute these associations [16], [38]. Prospective, well conducted studies are lacking and many of the retrospective studies have small numbers with a significant bias making definitive conclusions and comparisons difficult [39].

Most of the studies to date have looked at the relation between psychiatric diagnoses and HG rather than a broader range of mood, cognitive and behavioural states that may be applicable to a wider group of patients. Few studies have examined the association between anxiety in pregnancy and HG with one study showing that women with post traumatic stress disorder had 3.9 times the odds of been hospitalised for HG [40]. Simpson et al. demonstrated that women suffering from HG were more likely to suffer from somatisation, anxiety, psychoticism and obsessive compulsive symptoms. However, definitive conclusions are limited by small numbers [41]. Similarly, previous studies investigating the association between HG and preterm birth are also limited by the quality of the preterm birth data (failing to separate spontaneous and iatrogenic preterm birth) resulting in conflicting results which are difficult to decipher. The prospective nature of the SCOPE study allows us for the first time to conclusively demonstrate an association between spontaneous preterm birth and severe HG. In contrast to other large epidemiological studies, we have not demonstrated any differences in infant sex ratio in women with HG [17], [18], [19].

This paper is the first to provide a prospective cognitive, behavioural and emotional assessment of women who have suffered from HG. Although, depressed mood, perceived stress and the tendency to restrict activity in response to pregnancy resolved following cessation of vomiting, women with HG continued to display elevated anxiety scores potentially implicating high levels of anxiety as a risk factor for HG.

This paper has also examined a range of secondary outcomes and refutes studies which have associated HG with SGA while supporting the association of severe HG with spontaneous preterm birth. The prospective nature of our study has allowed us to examine clearly the association of varying degrees of severity of HG on pregnancy outcomes and highlighted differences depending on the degree of illness. This paper highlights the psychological impact of illness in pregnancy and demonstrates that women with HG, particularly severe HG requiring hospitalisation, display cognitive, behavioural and emotional difficulties. Because psychological factors such as depression have been linked to adverse pregnancy outcomes, providing more support for women with HG may help alleviate their feelings of stress and distress and be important for good antenatal care. In addition, helping women to manage anxiety may also help alleviate the HG symptoms.
